# Antihyperlipidemic Properties of Novel *N*-(Benzoylphenyl)-5-substituted-1*H*-indole-2-carboxamides in Triton WR-1339-Induced Hyperlipidemic Rats

**DOI:** 10.3390/molecules16108292

**Published:** 2011-09-29

**Authors:** Yusuf Al-Hiari, Ghassan Shattat, Tariq Al-Qirim, Waseem El-Huneidi, Ghassan Abu Sheikha, Suhair Hikmat

**Affiliations:** 1 Faculty of Pharmacy, University of Jordan, Amman-11942, Jordan; 2 Faculty of Pharmacy, Al-Zaytoonah Private University, Amman-11733, Jordan

**Keywords:** Triton WR-1339-induced hyperlipidemic rats, N-(benzoylphenyl)-5-methoxy-1*H*-indole-2-carboxamide, N-(Benzoylphenyl)-5-chloro-1*H*-indole-2-carboxamide, hypo-lipidemic activity

## Abstract

In the search for new potential antihyperlipidemic agents, the present study focuses on the synthesis of novel N-(benzoylphenyl)-5-substituted-1*H*-indole-2-carboxamides (compounds **8**-**12**, **15**, **16**, **18**) and investigating their antihyperlipidemic activity using Triton WR-1339-induced hyperlipidemic rats as an experimental model. Hyperlipidemia was developed by intraperitoneal injection of Triton WR-1339 (250 mg/kg body weight). The tested animals were divided into normal control (NCG), hyperlipidemic (HG), compound **8**, **9**, **15**, **16**, **18**- and bezafibrate treated groups. At a dose of 15 mg/kg body weight, compounds **9**, **16**, **18** and bezafibrate (100 mg/kg) significantly (*p* < 0.0001) reduced elevated plasma triglycerides levels after 12 h compared to the hyperlipidemic control group. However, only the group treated with compounds **9**, **16** and **18** showed an obviously significant (*p* < 0.001) reduction in plasma total cholesterol levels after 12 h compared to the hyperlipidemic control group. Moreover, high density lipoprotein-cholesterol levels were significantly (*p* < 0.0001) increased in all treated groups after 12 h compared to the hyperlipidemic control group, except for compounds **8** and **15** which revealed inactive. It is therefore reasonable to assume that compounds **9**, **16 **and **18** may have potential in the treatment of hyperlipidemia.

## 1. Introduction

Cardiovascular diseases are the most common cause of death in developed countries [1]. Hyperlipidemia is defined as elevation of one or more of the plasma lipids, including triglycerides, cholesterol, cholesterol esters and phospholipids [2]. This pathological condition has been ranked as one of the most important risk factors contributing to the occurrence and severity of cardiovascular diseases [3,4]. Many clinical trials have demonstrated that increase in plasma total cholesterol (TC) and triglycerides (TG) levels are implicated in the development of atherosclerosis [5,6].

In the early 1950s, it was noted that intravenous injection of certain nonionic detergents resulted in milky serum that lasted up to 48 h [7]. This was shown later to be due to the inhibition of TG hydrolysis by lipoprotein lipase (LPL) [8]. Since then, lipolysis inhibition has been used to determine hepatic TG production rates, with Triton WR-1339 (also known as tyloxapol). Triton WR-1339-induced hyperlipidemic rats are widely used as a model to screen for or to differentiate the mechanism of action of potential hypolipidemic agents [9,10,11].

Fibrates and their derivatives are group of drugs, which have been widely used to treat hyperlipoproteinemia, in particular cases with elevated TG. Bezafibrate is a member of this class which is a commercially available drug [12]. The major pharmacological mechanism of fibrates, including bezafibrate, is supposed to be an increased hydrolysis of TG by the induction of lipoprotein lipase and reduction of apolipoprotein C-III synthesis [13]. In spite of extensive research and development of numerous drugs, the anti-hyperlipidemic therapy is still underprivileged in term of efficiency, safety and thorough knowledge of the exact mechanisms.

**Figure 1:**
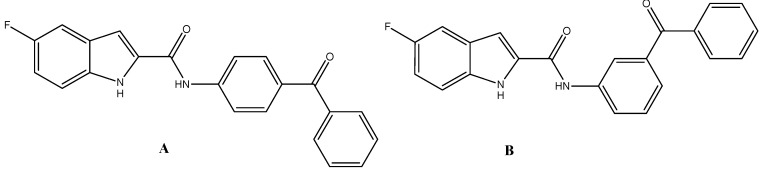
N-(Benzoylphenyl)-5-fluoro-1H-indole-2-carboxamide derivatives (**A**, **B**) with significant hypolipidemic effects.

During the last decade, a lot of attention has been given to studies focused on the synthesis of indole containing agents and their pharmacological activities. From these studies, which include our previously published work, it was found that compounds containing the indole-2-carboxamodes have a promising potential effects as lipid-lowering agents [14,15,16,17,18,19,20]. Previous work by our group revealed that N-(benzoylphenyl)-5-fluoro-1*H*-indole-2-carboxamide derivatives (Figure 1) exhibited significant hypolipidemic effects [19].

**Scheme 1:**
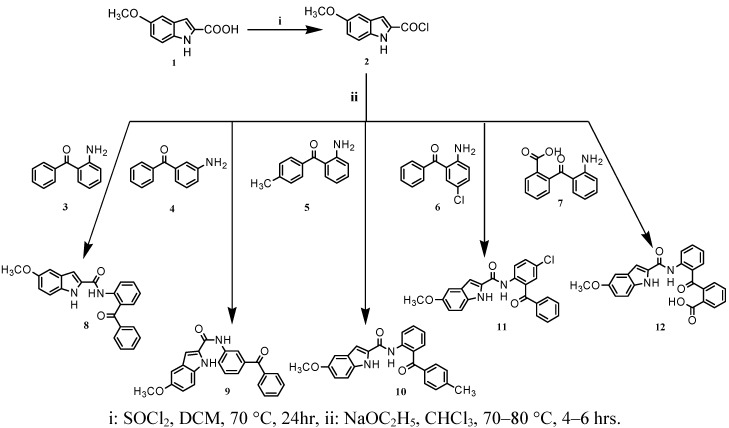
Preparation of N-(benzoylphenyl)-5-methoxy-1*H*-indole-2-carboxamides **8**-**12** starting from 5-methoxyindole-2-carbonyl chloride (**2**).

**Scheme 2:**
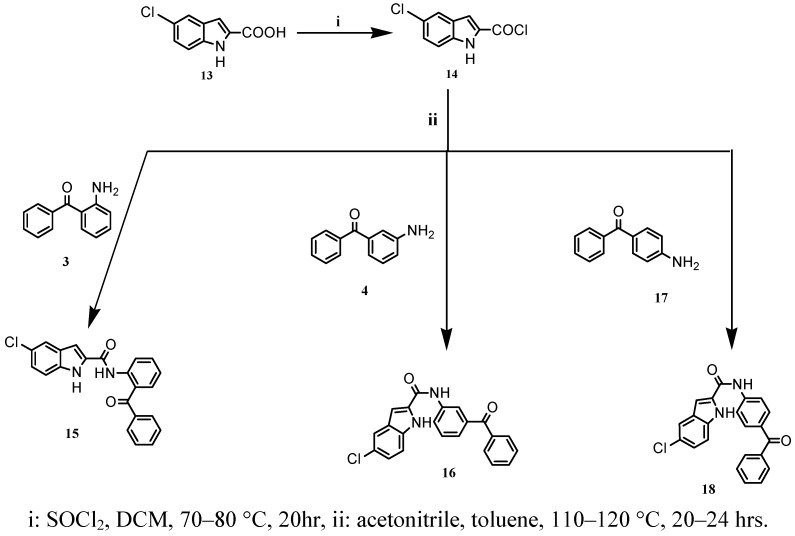
Preparation of N-(benzoylphenyl)-5-chloro-1*H*-indole-2-carboxamides **15**, **16**, **18** starting from 5-chloroindole-2-carbonyl chloride (**14**).

Given the importance in fighting hyperlipidemia, which is the prevalent risk of cardiovascular diseases, the present study focuses on the synthesis (Scheme 1 and Scheme 2) and pharmacological evaluation of novel N-(benzoylphenyl)-5-substituted-1*H*-indole-2-carboxamide derivatives from 5-methoxy- and 5-chloroindole carboxamides (compounds **8**-**12**, **15**, **16**, **18**). These compounds were then screened as models for their lipid-lowering effect.

## 2. Results and Discussion

### 2.1. Chemical Studies

In the present study, we aimed to evaluate the hypolipidemic activity of Novel N-(Benzoylphenyl)-5-substituted-1*H*-indole-2-carboxamide derivatives, using Triton WR-1339-induced hyperlipidemic rats, which have been widely used as a model for screening the lipid lowering effect of synthesized and naturally derived compounds [9,10,21,22,23].

5-Methoxyindole-2-carboxylic acid (**1**) was treated with thionyl chloride and dichloromethane. The mixture was refluxed at 70 °C for one day, furnishing the 5-methoxyindole-2-acylchloride (**2**) as a pure white solid and in high yield. The acid chloride was next refluxed with aminobenzophenones **3**-**7** in chloroform using excess sodium ethoxide as a catalyst (Scheme 1). The reaction was monitored by thin layer chromatography (TLC) revealing many side products if the reaction time exceeded 10 hours. It was found that heating the mixture at 70 °C for 4 to 6 hours gave low yields, but isolable fairly pure compounds. Purification using column chromatography gave the final products **8**-**12** in moderate to low amounts but high purity, aside from compound **12** all these compounds were successfully prepared in acceptable yields upon chromatography separation. Although alternative procedures gave higher yields of targets, our main interest was to collect sufficient amounts for pharmacological screening.^ 1^H- NMR, ^13^C-NMR, IR, elemental and MS analysis were used in structural elucidation and confirmed the proposed structures of the target compounds.

The preparation of the 5-chloroindole derivatives **15**, **16** and **18** was started by preparing 5-chloroindole-2-carbonyl chloride (**14**), which was obtained from 5-chloroindole-2-carboxylic acid (**13**) by acylation with thionyl chloride in DCM at 70–80 °C. Coupling of the acid chloride **14** with the corresponding benzophenone amine derivatives **3**, **4**, **17** in the presence of acetonitrile and toluene at 100–110 °C gave the target compounds (Scheme 2). The precipitated products were filtered and recrystallized to afford pure target compounds in satisfactory yields.

### 2.2. Pharmacological Studies

#### 2.2.1. Induction of Hyperlipidemia by Triton WR-1339

In this model, parenteral administration of Triton WR-1339 to adult rats could induce hyperlipidemia. The peak plasma total cholesterol and triglyceride levels were reached at 20 h followed by a decline to normal values [18,19,20,24]. In the current study, the same model showed a similar pattern of lipid profile changes 12 h after Triton WR-1339 administration.

The plasma total cholesterol (TC), triglyceride (TG), high-density lipoprotein-cholesterol (HDL-C), and low-density lipoprotein-cholesterol (LDL-C) levels of all groups treated after 12 h are shown in Table 1. Triton WR-1339 caused a significant increase in plasma TC, TG and LDL-C (*p* < 0.0001) levels in the hyperlipidemic control group (HG), 12 h after Triton WR-1339 administration in comparison with the normal control group (NCG).

In fact, the increase of plasma total cholesterol concentration in the HG was 113% after 12 h as compared to the NCG. The triglyceride level in the HG was also increased by 1064% after 12 h. At the same time the LDL-C level in HG was elevated by 98% after 12 h as compared to the NCG, while a significant decrease (39%, *p* < 0.0001) in the HDL-C was noticed 12 h after Triton WR-1339 injection.

**Table 1:** Effect of the novel compounds **8**, **9**, **15**, **16**, **18** and bezafibrate on plasma lipid levels in Triton WR-1339-induced hyperlipemic rats after 12 h.

Lipid profile	TC (mg/dl)	TG (mg/dl)	HDL-C(mg/dl)	LDL-C (mg/dl)
**NCG**	107 ± 0.76	131 ± 1.52	50.3 ± 0.40	31.1 ± 0.47
**HG**	228 ± 3.32^ b^	1526 ± 8.06^ b^	30.6 ± 0.92^ b^	61.6 ± 0.88^ b^
**C8**	227 ± 3.53	1544 ± 3.38	28.0 ± 0.73	65.9 ± 0.50
**C9**	112 ± 1.09^ a^	85 ± 1.60^ b^	59.2 ± 0.88^ b^	23.5 ± 0.53^ a^
**C15**	220 ± 4.63	1566 ± 4.44	29.9 ± 0.33	63.6 ± 0.42
**C16**	109 ± 2.10^ a^	96 ± 0.70^ b^	61.0 ± 0.56^ b^	24.9 ± 0.23^ a^
**C18**	106 ± 0.99^ a^	103 ± 2.43^ b^	63.1 ± 0.31^ b^	21.2 ± 0.31^ a^
**BF**	221 ± 2.40	132 ± 0.64^ b^	48.5 ± 0.48^ b^	60.9 ± 0.79

Values are expressed as means ± SD from eight animals in each group. HG, hyperlipidemic control group; C8, compound **8** + 4% DMSO; C9, compound **9** + 4% DMSO; C15, compound **15** + 4% DMSO; C16, compound **16** + 4% DMSO; C18, compound **18** + 4% DMSO; BF, bezafibrate + 4% DMSO; TC, total cholesterol; TG, triglyceride; HDL-C, high-density lipoprotein-cholesterol; LDL-C, low-density lipoprotein-cholesterol. Compounds **8**, **9**, **15**, **16**, **18** and BF are compared with HG. HG is compared with NCG. ^a^ *p* < 0.001, ^b^ *p* < 0.0001.

#### 2.2.2. Effect of Compounds **8**, **9**, **15**, **16**, **18** and Bezafibrate on Rat Plasma Lipid Profile

The plasma TC, TG, HDL-C, and LDL-C levels of bezafibrate (BF), and compound **8**-, **9**-, **15**-, **16**- and **18**-treated rats after 12 h are shown in Table 1. Importantly, the elevated plasma TG levels produced by Triton WR-1339 administration were significantly (*p* < 0.0001) concealed by 91%, 94%, 93% and 93% in BF, compound **9**-, **16**- and **18**-treated rats, respectively, with respect to hyperlipidemic control (HG).

The HDL-C levels were significantly increased after 12 h by 93%, 99%, 106% and 58% (*p* < 0.0001) in rats treated with compounds **9**, **16**, **18** and **BF**, respectively, compared to hyperlipidemic control HG (Table 1).

Rats treated with compounds **9**, **16** and **18** showed a significant (*p* < 0.001) reduction in plasma total cholesterol levels after 12 h (Table 1). In fact, it was found that the total cholesterol level was reduced by 51%, 52% and 54% after 12 h in compound **9**-, **16**- and **18**-treated rats respectively, compared to HG-treated rats. After 12 h of treatment, LDL-C levels were lowered by 62%, 60% and 66% (*p* < 0.001) in compound **9**-, **16**- and **18**-treated rats, respectively, compared to the hyperlipidemic control group, HG (Table 1). No significant differences in TC, TG, HDL-C, and LDL-C levels were observed in compound **8-** and **15**-treated rats compared to HG-treated rats.

Our results show that the hyperlipidemia induced by Triton WR-1339 was significantly suppressed with compounds **9**, **16**, **18** and bezafibrate in comparison with the control group after 12 h, and it was observed that the hypolipidemic effects of compounds **9**, **16** and **18** were obviously higher for triglycerides than for cholesterol. These results could be explained by understanding that the large increase in plasma cholesterol and triglycerides after Triton WR-1339 administration resulted mostly from an increase of the secretion of very low-density lipoprotein VLDL by the liver. In VLDL, the triglycerides proportion is several times greater than that of cholesterol and this is accompanied by a strong reduction of VLDL and LDL catabolism [25]. This result suggests that the compounds are partially able to restore the catabolism of lipoproteins.

In addition, a noticeable reduction in plasma total cholesterol was observed in the compound **9-**, **16-** and **18**-treated groups. This reduction was associated with a decrease of its LDL fraction, which is considered as main coronary heart disease. This result suggests that the cholesterol-lowering activity of these novel compounds can be a direct result of the enhancement of LDL catabolism through hepatic receptor [23].

In addition, HDL-C levels were significantly increased with compounds **9**, **16** and **18** after 12 h of Triton WR-1339 administration, which may have a protective role against atherogenesis [26]. HDL enhances the mobilization of triglycerides and cholesterol from plasma to liver where it is catabolised and secreted in the form of bile acids [27].

It is important to point out that compounds **10**, **11** and **12** were not biologically tested because they are derivatives of compound **8** and **15** (2-aminobenzophenone) which showed no activity in this model. Build up on previous data [19], it can be concluded that C-5 substitution on the indole system has no significant effect on hypolipidemic activity. However, active hypolipidemic compounds which involve N-(3-or 4-benzoylphenyl)-1*H*-indole-2-carboxamide (**9**, **16**, **18**) might imply that active hits favor an extended structure.

In sum, compounds **9**, **16**, **18** were shown to improve the lipid profile in Triton-induced hyperlipidemic rats. These findings are compatible with our previous published data, which confirm that compounds possess indole-2-carboxamide nuclei have lipid lowering effects [11,18,19,20]. The results are highly promising, but more studies are necessary to understand the exact mechanism of action of these novel compounds as antihyperlipidemic agents and to clarify their structure-activity relationships.

## 3. Experimental

### 3.1. General

Chemicals and reagents used were obtained from Aldrich Chemicals UK Ltd and Acros Ltd (UK). Solvents and reagents were of general grade unless stated otherwise. Melting points were measured using a Gallenkamp melting point apparatus and are uncorrected. ^1^H-NMR and ^13^C-NMR spectra were collected on a Varian Oxford NMR-300 MHz or Bruker Ultra shield 300 MHz spectrometer. The samples were dissolved in DMSO-d_6_. Mass spectrometry was performed using a Bruker Apex-IV mass spectrometer with an electrospray interface (ESI) (Bruker, Bremen, Germany). Infrared spectra were recorded using a Shimadzu IR Affinity-1 spectrophotometer. The samples were dissolved in CHCl_3_ and analyzed as thin solid films using NaCl plates. Analytical thin layer chromatography (TLC) was carried out using pre-coated aluminum plates and visualized by UV light (λ = 254 and/or 360 nm). Elemental analysis was performed using a EuroVector 2000 elemental analyzer (Milan, Italy).

*5-Methoxyindole-2-carbonyl chloride* (**2**). A mixture of 5-methoxy-indole-2-carboxylic acid (**1**, 0.5 g, 2.6 mmol) and thionyl chloride (5 mL) dissolved in dry dichloromethane (DCM, 30 mL). The reaction mixture was stirred and refluxed for 24 h at 70 °C till the completion of the reaction. Solvent was evaporated under vacuum, then chloroform was added twice and evaporated again to dryness in order to remove the excess of thionyl chloride present and thus afford compound **2** as a white solid (0.49, 89%). The product was used for preparation of all amide derivatives.

*N-(2-Benzoylphenyl)-5-methoxy-1H-indole-2-carboxamide* (**8**). 5-Methoxyindole-2-carbonyl chloride (**2**, 0.50 g, 2.4 mmol) was added to a solution of 2-aminobenzophenone (**3**, 0.94 g, 4.8 mmol) and sodium ethoxide (NaOC_2_H_5_, 0.32 g, 4.7 mmol) in chloroform (25 mL). The mixture was refluxed for 6 h at 80 °C then the reaction mixture was filtered, and the solid recrystallized from CHCl_3_ to afford a yellow solid (**8**, 0.65 g, 74%); m.p.: 230 °C; ^1^H-NMR (DMSO-d_6_): δ = 11.66, 11.65 (2br s, 1H, NH, rotamers), 10.99, 10.97 (2br s, 1 H, NH, rotamers), 7.89-7.84 (m, 1H, Ar-H), 7.7-7.72 (m, 2H, Ar-H), 7.6-7.55 (m, 2H, Ar-H), 7.5-7.39 (m, 3H, Ar-H), 7.3-7.23 (m, 2H, Ar-H), 7.18 (m, 1H, Ar-H), 7.08 (m, 1H, Ar-H), 6.86-6.81 (m, 1H, Ar-H), 3.76, 3.73 (2br s, 3H, OCH_3_, rotamers); ^13^C-NMR (DMSO-d_6_): δ = 196.05 (CO ketone), 159.83 (CONH), 154.28, 137.72, 137.18, 132.95, 132.61, 132.53, 131.65, 130.86, 130.61, 130.24, 128.64, 127.65, 124.36, 124.04, 115.69, 113.65, 104.20, 102.44, 55.70 (OCH_3_) ppm; IR (thin film): ν = 3449, 3291, 1667, 1628, 1582, 1535, 1450, 1261, 1219, 1157, 1030, 940, 845, 755 cm^−1^; MS (ESI, positive mode): *m/z* [M+H]^+^ 371.13968 (C_23_H_19_N_2_O_3_ requires 371.13957); Anal. Calcd for C_23_H_18_N_2_O_3_: C, 74.58; H, 4.90; N, 7.56. Found: C, 74.69; H, 4.79, N, 7.60.

*N-(3-Benzoylphenyl)-5-methoxy-1H-indole-2-carboxamide* (**9**). 5-Methoxyindole-2-carbonyl chloride (**2**, 0.40 g, 1.9 mmol) was added to a solution of 3-aminobenzophenone (**4**, 0.75 g, 3.8 mmol) and sodium ethoxide (NaOC_2_H_5_, 0.26 g, 3.8 mmol) in chloroform (25 mL). The mixture was refluxed for 4 h at 80 °C. The organic layer was removed by evaporation under reduced pressure and the residue was separated by column chromatography using chloroform/methanol (99:1) as eluent to afford an off-white solid (**9**, 50 mg, 7%); m.p.: 200 °C; ^1^H-NMR (DMSO-d_6_): δ = 11.52 (br s, 1H, NH), 10.41 (br s, 1H, NH), 8.25 (br s, 1H, Ar-H), 8.18 (d, *J* = 8.2 Hz, 1H, Ar-H), 7.79 (d,d, *J* = 8.4, 1.3 Hz, Ar-H), 7.68-7.73 (m, 1H, Ar-H), 7.65-7.55 (m, 3H, Ar-H), 7.48 (d, *J* = 7.7 Hz, 1H, Ar-H), 7.36 (d,d, *J* = 8.3, 2.0 Hz, 2H, Ar-H), 7.15 (d, *J* = 2.1 Hz, 1H, Ar-H), 6.89 (d,d, *J* = 8.9, 2.3 Hz, 1H, Ar-H), 3.78 (s, 3H, OCH_3_); ^13^C-NMR (DMSO-d_6_): δ = 196.13 (CO ketone), 160.38 (CONH), 154.36, 139.70, 137.88, 137.55, 133.16, 132.72, 131.86, 130.11, 129.53, 129.05, 127.78, 125.05, 124.39, 121.54, 115.75, 113.72, 104.34, 102.58, 55.75 (OCH_3_) ppm; IR (thin film): ν = 3356, 3291, 2936, 1643, 1585, 1543, 1431, 1281, 1258, 1092, 1030, 890, 790, 740 cm^−1^; MS (ESI, positive mode): *m/z* [M+Na]^+^ 393.12096 (C_23_H_18_N_2_NaO_3_ requires 393.12151); Anal. Calcd for C_23_H_18_N_2_O_3_: C, 74.58; H, 4.90; N, 7.56. Found: C, 74.62; H, 4.92, N, 7.60.

*5-Methoxy-N-[2-(4-methylbenzoyl)phenyl]-1H-indole-2-carboxamide* (**10**). 5-Methoxyindole-2-carbonyl chloride (**2**, 0.40 g, 1.9 mmol) was added to a solution of 2-amino-4'-methyl benzophenone (**5**, 0.80 g, 3.8 mmol) and sodium ethoxide (NaOC_2_H_5_, 0.26 g, 3.8 mmol) in chloroform (30 mL). The mixture was refluxed for 4 h at 80 °C. The organic layer was removed by evaporation under reduced pressure and the residue was purified by column chromatography using cyclohexane/ ethyl acetate (8:2) to afford a yellow solid (**10**, 80 mg, 11%); m.p.: 210 °C; ^1^H-NMR (DMSO-d_6_): δ = 11.62 (br s, 1H, NH), 10.85 (br s, 1H, NH), 7.91 (m, *J* = 8.1 Hz, 1H, Ar-H), 7.68-7.56 (m, 3H, Ar-H), 7.50-7.44 (m, 1H, Ar-H), 7.40-7.23 (m, 4H, Ar-H), 7.13 (m, 2H, Ar-H), 6.87 (d,d, *J* = 8.2, 2.4 Hz, 1H, Ar-H), 3.76 (s, 3H, OCH_3_), 2.37 (s, 3H, CH_3_); ^13^C-NMR (DMSO-d_6_): δ = 196.08 (CO ketone), 159.85 (CONH), 154.33, 143.41, 137.35, 135.14, 132.67, 132.60, 131.67, 131.10, 130.47, 130.40, 130.29, 129.49, 129.25, 127.71, 124.28, 123.87, 115.73, 113.66, 103.79, 102.50, 55.71 (OCH_3_), 21.61 (CH_3_) ppm; IR (thin film): ν = 3294, 2959, 1667, 1578, 1524, 1447, 1261, 1215, 1022, 930, 799, 752 cm^−1^; MS (ESI, positive mode): *m/z* [M+Na]^+^ 407.13661 (C_24_H_20_N_2_NaO_3_ requires 407.13716); Anal. Calcd for C_24_H_20_N_2_O_3_: C, 74.98; H, 5.24; N, 7.29. Found: C, 74.95; H, 5.20, N, 7.31.

*N-[2-(Benzoyl-4-chlorophenyl)]-5-methoxy-1H-indole-2-carboxamide* (**11**). 5-Methoxyindole-2-carbonyl chloride (**2**, 0.40 g, 1.9 mmol) was added to a solution of 2-amino-5-chlorobenzophenone (**6**, 0.88 g, 3.8 mmol) and sodium ethoxide (NaOC_2_H_5_, 0.26 g, 3.8 mmol) in chloroform (35 mL). The mixture was refluxed for 4 h at 80 °C. The organic layer was removed by evaporation under reduced pressure and the residue was purified by column chromatography using cyclohexane/ethyl acetate (85:15) to afford a yellow solid (**11**, 60 mg, 8%); m.p.: 200 °C; ^1^H-NMR (DMSO-d_6_): δ = 11.56 (br s, 1H, NH), 10.69 (s, 1H, NH), 7.78-7.70 (m, 4H, Ar-H), 7.62-7.57 (m, 1H, Ar-H), 7.49 (d,d, *J* = 7.6, 7.1 Hz, 2H, Ar-H), 7.43 (d, *J* = 1.6 Hz, 1H, Ar-H), 7.26 (d, *J* = 8.9 Hz, 1H, Ar-H), 7.09 (m, *J* = 1.9 Hz, 2H, Ar-H), 6.85 (d,d, *J* = 8.9, 2.4 Hz, 1H, Ar-H), 3.76 (s, 3H, OCH_3_); ^13^C-NMR (DMSO-d_6_): δ = 195.60 (CO ketone), 159.87 (CONH), 154.31, 136.97, 135.62, 133.32, 132.66, 132.06, 131.20, 130.25, 129.80, 128.75, 128.60, 127.60, 126.29, 115.85, 113.66, 104.15, 102.45, 55.70 (OCH_3_) ppm; IR (thin film): ν = 3298, 2924, 2851, 1659, 1628, 1578, 1531, 1508, 1458, 1288, 1246, 1219, 1035, 966, 840, 805, 755 cm^−1^; MS (ESI, positive mode): *m/z* [M+Na]^+^ 427.08199 (C_23_H_17_ClN_2_NaO_3_ requires 427.08254); Anal. Calcd for C_23_H_17_ClN_2_O_3_: C, 68.23; H, 4.23; N, 6.92. Found: C, 68.45; H, 4.32, N, 6.90.

*2-(2-{[(5-Methoxy-1H-indole-2-yl)carbonyl]amino}benzoyl)benzoic acid* (**12**). 5-Methoxyindole-2-carbonyl chloride (**2**, 0.50 g, 2.4 mmol) was added to a solution of 2-aminobenzophenone-2'-carboxylic acid (**7**, 1.15 g) and NaOC_2_H_5_ (0.32 g, 4.8 mmol) in chloroform (25 mL). The mixture was refluxed for 4 h at 80 °C. The organic layer was removed by evaporation under reduced pressure and the residue was purified by column chromatography using cyclohexane/ethyl acetate (75:25) to afford a yellow-orange solid (**12**, 60 mg, 6%); m.p.: 240 °C. The low amount collected and its decomposition precluded further analysis and biological screening.

*5-Chloroindole-2-carbonyl chloride* (**14**)*.* A mixture of 5-chloroindole-2-carboxylic acid (**13**, 1 g, 5.11 mmol) and thionyl chloride (SOCl_2_) (3.6 mL) in dry DCM (20 mL) was added in a round bottomed flask. The reaction mixture was refluxed for 20 h at 70–80 °C till the completion of the reaction. Solvent was evaporated under vacuum, then DCM was added twice and evaporated again to dryness in order to remove the excess of thionyl chloride present, and thus afford acid chloride **14** (0.95 g, 86%) as a yellow precipitate; R_f_: 0.71 (cyclohexane/ethyl acetate, 7:3); m.p.= 209 °C; ^1^H-NMR (DMSO-d_6_): δ = 11.90 (s, 1H, NH-indole), 7.65 (d, *J* = 7.8 Hz, 1H, Ar-H), 7.65 (d, *J* = 7.8 Hz, 1H, Ar-H), 7.47 (d, *J* = 8.1 Hz, 1H, Ar-H), 7.26 (dd, *J* = 7.2, 7.8 Hz, 1H, Ar-H), 7.16 (s, 1H, Ar-H), 7.07 (dd, *J* = 7.5, 7.2 Hz, 1H, Ar-H); ^13^C-NMR (DMSO-d_6_): δ = 162.21 (CO), 137.89, 127.54, 127.20, 125.09 (CH-Ar), 122.51 (CH-Ar), 120.63 (CH-Ar), 113.05 (CH-Ar), 108.23 (CH-Ar) ppm; IR (thin film): ν = 3368, 3294, 1679, 1645, 1592, 1547, 1284, 1215, 851, 790 cm^−^^1^.

*N-[2-Benzoylphenyl]-5-chloro-1H-indole-2-carboxamide* (**15**). 5-Chloroindole-2-carbonyl chloride (**14**, 0.40 g, 1.86 mmol) was added to a solution of 2-aminobenzophenone (**3**, 1.50 g, 7.6 mmol) in a mixture of acetonitrile (20 mL) and dry toluene (20 mL). The mixture was refluxed for 24 h at 110 °C, then cooled. The organic layer was removed by evaporation under reduced pressure and the residue was crystallized using chloroform/methanol to afford a yellow solid (**15**, 0.36 g, 52%); R_f_: 0.51 (cyclohexane/ethyl acetate, 7:3); m.p.: 260 °C; ^1^H-NMR (DMSO-d_6_): δ = 11.94 (br s, 1H, NH), 10.88 (br s, 1H, NH), 7.85 (d, *J* = 7.92 Hz, 1H, Ar-H), 7.75 (m, 2H, Ar-H), 7.71-7.57 (m, 3H, Ar-H), 7.50-7.36 (m, 2H, Ar-H), 7.41-7.24 (m, 3H, Ar-H), 7.23-7.13 (m, 2H, Ar-H); ^13^C-NMR (DMSO-d_6_): δ = 196.32 (CO-ketone), 158.87 (CONH), 143.21, 138.23, 137.26, 135.11, 132.47, 131.41, 131.01, 130.37, 129.21, 127.38, 124.59, 124.42, 124.13, 121.31, 113.39, 104.16 ppm; IR (thin film): ν = cm^−1^; 3344, 2955, 1671, 1575, 1524, 1261, 1215, 1022, 930, 799; HRMS (ESI, negative mode): *m/z* (M^+^ - H^+^) 373.07422 (C_22_H_14_ClN_2_O_2_) requires 373.07438; Anal. Calcd for C_22_H_15_ClN_2_O_2_: C, 70.50; H, 4.03; N, 7.74. Found: C, 70.82; H, 4.11; N, 7.22.

*N-[3-Benzoylphenyl]-5-chloro-1H-indole-2-carboxamide* (**16**). 5-Chloroindole-2-carbonyl chloride (**14**, 0.50 g, 2.34 mmol) was added to a solution of 3-aminobenzophenone (**4**, 1.25 g, 6.34 mmol) in acetonitrile (15 mL) and dry toluene (20 mL). The mixture was refluxed for 24 h at 110 °C, then cooled. The organic layer was removed by evaporation under reduced pressure and the residue was crystallized using chloroform/methanol to afford a yellow crystals (**16**, 0.55 g, 63%); R_f_: 0.37 (cyclohexane/ethyl acetate, 7:3); m.p.: 255 °C; ^1^H-NMR (DMSO-d_6_): δ = 12.01 (br s, 1H, NH), 10.54 (s, 1H, NH), 8.25 (s, 1H, Ar-H), 8.18 (d, *J* = 7.97 Hz, 1H, Ar-H), 7.84-7.72 (m, 3H, Ar-H), 7.69 (d, *J* = 7.20 Hz, 1H, Ar-H), 7.65-7.53 (m, 3H, Ar-H), 7.50 (d, *J* = 4.75 Hz, 1H, Ar-H), 7.46 (br s, 2H, Ar-H), 7.24 (dd, *J* = 8.61, 8.61, 1.6 Hz, 1H, Ar-H); ^13^C-NMR (DMSO-d_6_): δ = 196.07 (CO-ketone), 160.07 (CONH), 139.52, 137.90, 137.53, 135.75, 133.16 (CH-Ar), 133.10, 130.11 (CH-Ar), 129.58 (CH-Ar), 129.05 (CH-Ar), 128.48, 125.23 (CH-Ar), 124.93, 124.51(CH-Ar), 124.43 (CH-Ar), 121.62 (CH-Ar), 121.35 (CH-Ar), 114.48 (CH-Ar), 104.18 (CH-Ar) ppm; IR (thin film): ν = 3445, 2999, 1665, 1565, 910, 812 cm^−1^; HRMS (ESI, negative mode): *m/z* (M^+^ - H^+^) 373.07441 (C_22_H_14_ClN_2_O_2_) requires 373.07438; Anal. Calcd for C_22_H_15_ClN_2_O_2_: C, 70.50; H, 4.03; N, 7.47. Found: C, 69.99; H, 4.10; N, 7.19.

*N-[4-Benzoylphenyl]-5-chloro-1H-indole-2-carboxamide* (**18**). 5-Chloroindole-2-carbonyl chloride (**14**, 0.51 g, 2.35 mmol) was added to a solution of 3-aminobenzophenone (**17**, 1.24 g, 6.33 mmol) in acetonitrile (15 mL) and dry toluene (20 mL). The mixture was refluxed for 20 h at 110 °C, then cooled. The organic layer was removed by evaporation under reduced pressure and the residue was crystallized using chloroform/methanol to afford yellow crystals (**18**, 0.35 g, 39%); R_f_: 0.38 (cyclohexane/ethyl acetate, 7:3); m.p.: 256 °C; ^1^H-NMR (DMSO-d_6_): δ = 11.98 (br s, 1H, NH), 10.58 (s, 1H, NH), 8.20 (s, 1H, Ar-H), 8.18 (d, *J* = 8.12 Hz, 1H, Ar-H), 7.84-7.65 (m, 4H, Ar-H), 7.63-7.51 (m, 4H, Ar-H), 7.45 (m, 2H, Ar-H), 7.24 (m, 1H, Ar-H); ^13^C-NMR (DMSO-d_6_): δ = 195.45 (CO-ketone), 159.87 (CONH), 139.45, 137.88, 137.78, 135.55, 132.98, 133.15, 130.01, 129.48, 128.99, 128.58, 125.20, 124.97, 124.34, 124.44, 121.63, 121.34, 114.48, 104.18 ppm; IR (thin film): ν = 3475, 2965, 1675, 1585, 1554, , 841 cm^−1^; HRMS (ESI, negative mode): *m/z* (M^+^ - H^+^) 373.07437 (C_22_H_14_ClN_2_O_2_) requires 373.07438; Anal. Calcd for C_22_H_15_ClN_2_O_2_: C, 70.50; H, 4.03; N, 7.47. Found: C, 70.47; H, 4.12; N, 7.34.

### 3.2. Animals and Treatments

Sixty four adult male Wistar rats, weighing 180–250 g, bred in the animal care centre of Faculty of Pharmacy, Al-Zaytoonah University, Amman, Jordan, were provided *ad libitum* access only to tap water throughout the experimental duration. Rats were maintained in a 12 h light-dark cycle under constant humidity (55 ± 15%) and (22 ± 2 °C). All experiments were performed in accordance with the Guidelines for Animal Welfare Committee of Al-Zaytoonah University.

### 3.3. Triton Model of Hyperlipidemia

Triton WR-1339 dissolved in dimethylsulfoxide (DMSO) and administered intraperitoneally to the rats at a dose of (250 mg/kg body weight) in order to induce hyperlipidemia [28,29].

### 3.4. Pharmacological Experimental Design

Overnight fasted rats were randomly divided into eight groups of eight animals each. The first group, serving as normal control group (NCG) received an intraperitoneal administration of normal saline; the second hyperlipidemic group (HG) received an intraperitoneal injection of Triton 4% DMSO. In the third, fourth, fifth, sixth and seventh groups, rats were intraperitoneally injected with Triton, followed by an intragastric administration of (1 mL) of compounds **8**, **9**, **15**, **16** and **18** (15 mg/kg body weight) dissolved in 4% DMSO. The last group (BF) was also intraperitoneally injected with Triton and intragastrically treated with bezafibrate (100 mg/kg body weight) dissolved in 4% DMSO [30,31]. After 12 h of treatments, animals were anaesthetized with diethyl ether and blood was collected. The blood samples were immediately centrifuged (3000 rpm for 10 min) and the plasma was used for lipid analysis by an enzymatic method with an automatic analyzer (Model Erba XL-300, Germany, Mannheim, Germany)

### 3.5. Statistical Analysis

Results were expressed as mean ± SD. Data obtained were analyzed using the Student’s t-test, and differences with *p* < 0.05 were considered statistically significant.
